# Story Retelling and Verbal Working Memory in Young Adults With a History of COVID-19: Cross-Sectional Study

**DOI:** 10.2196/71303

**Published:** 2025-08-05

**Authors:** Hyunsoo Yoo

**Affiliations:** 1 Department of Communication Science and Disorders, Baylor University, 1311 S 5th St, Waco, TX, 76706, United States, 1 (800) 229-5678

**Keywords:** COVID-19, story retelling, verbal working memory, young adults, cognitive, linguistic

## Abstract

**Background:**

The impact of the COVID-19 pandemic has primarily been studied in the context of language delays or developmental disorders in infants and children. However, the effects on young adults have received less attention. COVID-19 not only affects physical health but also cognitive and language functions, which is an emerging area of research. While previous studies have focused on developmental stages, the effects of COVID-19 on the language abilities of healthy young adults remain underexplored. This study aimed to investigate the impact of COVID-19 on the spoken language, particularly in story retelling and working memory, in young adults.

**Objective:**

This study aimed to investigate the effects of COVID-19 on memory-based story retelling and verbal working memory in young adults. Specifically, it examined whether there were group differences in story retelling and working memory performance between individuals with and those without a history of COVID-19, and whether verbal working memory predicted story retelling outcomes.

**Methods:**

The study involved 79 young adult participants, of whom 39 were in the non–COVID-19 group and 40 were in the COVID-19 group. Participants completed the Story Retelling Procedure (SRP) and a verbal working memory task. Story retelling performance was quantified using information units per minute (IUs/min), a measure of informativeness in story retelling. Working memory was assessed using the Alphabet Span Test.

**Results:**

Participants with COVID-19 produced fewer information units per minute (mean 0.53, SD 0.21) than those without COVID-19 (mean 0.63, SD 0.24; *P*=.049). No significant group differences were found in verbal working memory performance (*P*=.20). However, regression analysis showed that verbal working memory significantly predicted story retelling performance (*R*²=.064, *P*=.02), suggesting that individual differences in working memory capacity may contribute to discourse informativeness, regardless of COVID-19 history.

**Conclusions:**

Young adults with a history of COVID-19 exhibited reduced story retelling performance compared to those without a history of infection. In contrast, no significant differences were observed in verbal working memory performance between groups. Furthermore, verbal working memory scores significantly predicted story retelling performance, suggesting a functional link between these cognitive-linguistic domains. These findings suggest that story retelling performance may serve as a sensitive indicator of post–COVID-19 cognitive-linguistic changes in young adults.

## Introduction

COVID-19 is caused by SARS-CoV-2, and this virus is known to cause respiratory illness in humans. After the first case was reported in December 2019, the total cumulative cases worldwide reached 770,875,433 by October 3, 2023, according to the World Health Organization [1]. Since the introduction of vaccines, the number of cases has decreased dramatically.

While hospitalizations and severe cases of respiratory symptoms have decreased, the impact of COVID-19 on cognition has persisted. Numerous studies have reported cognitive dysfunction in individuals with a history of COVID-19. A recent systematic review article [2] reported that the results are inconsistent. Another study [3] found that age was a risk factor, while other study [4] found that middle-aged individuals were the most vulnerable population. Gender differences were also found [5] in cognitive impairment, and women tend to have higher cognitive impairment than men.

Regarding measures tested for the cognitive abilities, the most common neuropsychological screening tests were commonly used in studies [6,7], but an inconsistency was also found here: Quan et al [2] claimed that the timing of cognitive testing might be the reason for the inconsistency of the results. Other tasks were also used, such as the Digit Span Test-forward and backwards, Trail Making Test-parts A and B, Stroop Word reading and Colour Naming, Corsi backwards, Stroop test, Rey auditory verbal learning test, Free and Cued Selective Reminding Test, Rey–Osterrieth Complex Figure, and Boston Naming Test, Verbal [8-12]. One study [13] found that the severity of COVID-19 was related to poor performance in executive function tasks (Digit symbol, Trail Making Test B, and phonetic fluency). Akıncı et al [14] also found that COVID-19 affected global cognitive skills, which were evaluated using the Montreal Cognitive Assessment and Clock-Drawing Test; memory functions, evaluated using the Öktem Verbal Memory Processes Test; attention span, evaluated using the Digit Span Test; executive functions, evaluated using fluency tests, the Stroop test, and the Trail Making Test; visual perceptual skills, evaluated using the Rey Osterrieth Complex Figure test; and neuropsychiatric status, evaluated using the Neuropsychiatric Inventory.

Although some studies included verbal language tasks such as the Boston Naming Test, discourse-level spoken language, particularly story retelling, has not been examined in COVID-19–related cognitive research in relation to verbal working memory. Story retelling is a cognitively demanding task that engages multiple components of verbal working memory, including temporary storage, manipulation, and retrieval of linguistic information [15,16]. Successful retelling requires not only remembering the sequence of events [17] but also integrating details, maintaining coherence, and regenerating narrative structure [18], all of which rely on both working memory capacity ([19]) and attentional control [20,21].

Emerging research suggests that COVID-19 infection may affect higher-order cognitive functions, such as sustained attention, executive function, and verbal memory [9,22-24]. These functions are closely tied to narrative production abilities, making story retelling a sensitive measure for detecting subtle post–COVID-19 cognitive changes, particularly in young adults with no overt language deficits.

Some of the studies used working memory tasks, but the format of the working memory tasks was based on visual working memory. According to Baddeley’s [25, 26] working memory model, visual working memory and verbal working memory are 2 distinct cognitive systems. Visual working memory is responsible for storing information and manipulating visual information such as shapes, colours, and images. Verbal working memory handles language-based information and involves also temporarily storing information and manipulating verbal or auditory information such as words, sentences, and numbers. Therefore, the nature of the information process is different, and it is processed in different systems: the visuospatial sketchpad for visual working memory and the phonological loop for verbal working memory. Considering that spoken discourse production has been shown to be a sensitive clinical indicator of language processing deficits in people with atypical neurogenic language disorders (eg, aphasia) [16,27,28], it is imperative to investigate the impact of COVID-19 on spoken discourse performance.

There are different types of spoken discourse formats depending on how the tasks are administered, and there are mainly 2 different types of tasks, one of which is story retelling. In this format, the examiner read a story, and the participants were asked to retell the story immediately after hearing it. The story retelling type taxes memory capacity [16] and is affected by recency and/or primacy effect [29]. Another format is based on picture description. In this format, participants can rely on the picture in front of them; therefore, the task places lower cognitive demands on memory compared to the story retelling format. Yoo et al [16] found the correlation between the verbal working memory tasks with the retelling type of discourse production results in people with aphasia. This aligns with the connection between verbal working memory and various levels of language processing, as extensively documented in the literature [26,30-32].

Therefore, the primary goal of this study was to examine whether COVID-19 affects memory-based story retelling performance and verbal working memory. The previously reported correlation between performance on the Story Retelling Procedure (SRP) and verbal working memory were correlated based on was assessed [16]. If working memory has declined after COVID-19, it is reasonable to hypothesize that story retelling performance would also be affected by COVID-19. The following questions were investigated in this study:

1. Are there significant differences in measures of story retelling between individuals with and those without a history of COVID-19?
2. Are there significant differences in the verbal working memory between individuals with and those without a history of COVID-19?
3. Is there a predictive relationship between the story retelling and the verbal working memory scores? In other words, can the verbal working memory scores predict the story retelling scores?

## Methods

### Ethical Considerations

This study was approved by the institutional review board at Baylor University (approval number 1655082‐11). All participants provided verbal informed consent prior to participation. Some personally identifiable information was collected as part of the study. All identifiable data were removed prior to analysis, and the remaining materials were securely stored on a university-provided cloud platform with restricted access. Only approved research personnel had access to the data through institutional login credentials. No identifying details (such as names, initials, facial features, or other unique identifiers) are included in the manuscript or any submitted materials. All data are presented in a manner that ensures participant anonymity and privacy. No compensation was provided to the participants.

### Participants

In total, 79 young participants in their 20s with and those without a history of COVID-19 participated in this study virtually: (1) the non–COVID-19 group comprised 39 participants (age: mean 21.58, SD 1.24 years; years of education: mean 16.32, SD 0.97), and (2) the COVID-19 group comprised 40 participants (age: mean 21.38, SD 1.63 years; years of education: mean 15.60, SD 1.25; Table 1).

Participants in the non–COVID-19 group were recruited between November 11, 2020, and December 4, 2020, and self-reported not having a history of COVID-19. Participants in the COVID-19 group were recruited between April 13, 2021, and September 20, 2022, and provided self-reported information regarding time since infection and number of infections. All COVID-19–positive participants were nonhospitalized, and symptom severity ratings were not collected using a formal scale. Vaccination status was not obtained, and information regarding post–COVID-19 condition symptoms was not collected, as the data were obtained during the early stage of the pandemic, before the concept of post–COVID-19 condition had been clearly defined. Table 2 provides a summary of COVID-19–related participant characteristics.

**Table 1.** Demographic information of participants.

Group	Participants, n	Values, mean (SD)
Age (years)		
Non–COVID-19 group	39	21.58 (1.24)
COVID-19 group	40	21.38 (1.62)
Total	79	—^a^
Years of education		
Non–COVID-19 group	39	16.32 (0.97)
COVID-19 group	40	15.61 (1.25)
Total	79	—

a Not applicable.

**Table 2.** Self-reported COVID-19 characteristics of the COVID-19 group (n=40).

Variable	Values
Time since infection (months)	1 infection: mean 256.6 (SD 199.28); 2 infections: mean 168.56.93 (SD 130.93)
Number of infections	1 infection: n=34 (85%); 2 infections: n=6 (15%)
Symptom severity	Not quantified; all nonhospitalized
Vaccination status	Not obtained
Post–COVID-19 symptoms	Not collected; data were obtained before the concept of post–COVID-19 was clearly defined

### Procedures

All data collection procedures were conducted virtually via Zoom (Zoom Video Communications). Each session lasted less than 1 hour per participant.

#### The SRP Task

The stories used in the SRP task were adapted from the Discourse Comprehension Test [33], which includes 10 stories that were normed and matched on key linguistic complexity variables such as number of words, sentence length, number of subordinate clauses, listening difficulty, and number of main ideas. The validity and reliability of the SRP as a discourse elicitation procedure have been established through validation studies [34,35], which demonstrated strong correlations across multiple linguistic domains with other established elicitation tasks, including picture descriptions and procedural narratives.

SRP participants listened to a short story, and they were asked to retell it immediately afterward. Three short stories were presented, and the averaged raw scores were used for the data analysis. The story information units were checked using the scoring sheet and correct information units per minute was calculated as an outcome variable.

#### The Working Memory Task

Each participant also completed Alphabet Span Task as a working memory task. In the task, each participant listened to a list of words and was asked to reorder the words in alphabetical order. The list of words was increased as the span increased. The total correct numbers of items were used as the outcome variable for this working memory test.

### Statistical Analysis

Statistical analyses for this project were conducted using Python (version 3.10), with the statsmodels and scipy packages for ANOVA and regression modeling. The dataset was assessed for normality, and due to skewed distributions, a natural log transformation (ln) was applied to the story retelling variable (information units per minute [IUs/min]). Two outcome variables were analyzed: (1) log-transformed IUs/min derived from the SRP task and (2) the total number of correct responses on the Alphabet Span task as a measure of verbal working memory.

To address the first research question, a 1-way between-subjects ANOVA was conducted to compare story retelling performance between the COVID-19 and non–COVID-19 groups. The dependent variable was the number of information units produced per minute, log-transformed to meet assumptions of normality.

## Results

### Overview

The results revealed a significant between-group difference (*F*_1,77_=3.99, *P*=.049), with the COVID-19 group producing fewer information units per minute (mean 0.53, SD 0.21) than the non–COVID-19 group (mean 0.63, SD 0.24). For the second research question, a separate 1-way ANOVA was conducted to compare verbal working memory performance between groups, measured by total number of correct items on the Alphabet Span task. The analysis indicated no significant difference between the COVID-19 (mean 16.21, SD 2.94) and non–COVID-19 (mean 15.30, SD 3.29) groups (*F*_1,77_=1.66, *P*=.20). To address the third research question, a simple linear regression was conducted to evaluate the extent to which verbal working memory performance predicted story retelling performance. The regression model was significant (*F*_1,77_=5.31, *P*=.02), and accounted for 6.4% of the variance in log-transformed IUs/min (*R*²=.064).

The regression equation was:

Story retelling performance (log IUs/min)=−0.493+0.014×verbal working memory performance (Alphabet Span Test: the total correct numbers of items).

The unstandardized regression coefficient (B=0.014) indicates that each additional point on the Alphabet Span Test resulted in a 0.014 increase in log-transformed IUs/min. This corresponds to an approximate 1.4% increase on the raw scale:


$$exp\left(0.014\right)=\;e^{\left\{0.014\right\}}\approx 1.014$$

This back-transformation should be interpreted cautiously. All statistical inferences were based on log-transformed values due to nonnormality in the original distribution. The 95% CI for the slope ranged from 0.002 to 0.026, suggesting a modest but statistically reliable association between verbal working memory and story retelling performance.

### Additional Exploratory Analyses

Additional exploratory analyses were conducted within the COVID-19 group to examine whether the number of days since infection was associated with performance on cognitive-linguistic tasks. Time since infection was not significantly associated with story retelling performance (*r*=.10; *P*=.57; *R*²=.009) or with verbal working memory scores (*r*=.04; *P*=.83; *R*²=.001). These results suggest that recovery time did not significantly account for performance variability in either task. All additional statistical analyses were conducted using Python (version 3.10) with the pandas, scipy.stats, and seaborn libraries. Exploratory correlation analyses were performed using the Python pearsonr function to assess linear associations between variables of interest, and simple linear regression was conducted using the linregress function.

## Discussion

### Principal Findings

This study aimed to investigate the impact of COVID-19 on spoken story retelling and verbal working memory and to examine the predictive relationship between the story retelling and the verbal working memory in young adults. The results found that the story retelling performance of the COVID-19 group was significantly lower than that of the non–COVID-19 group. However, despite this performance difference in story retelling, no significant difference was observed between the groups in verbal working memory. This highlights an interesting contrast, suggesting that while the COVID-19 group showed reduced story retelling performance, their verbal working memory capacity did not differ significantly from that of the non–COVID-19 group, indicating that other factors beyond memory might contribute to the observed differences in story retelling performance. In the results of regression analysis, the spoken story retelling performance, measured by %IUs/min, was significantly predicted by the verbal working memory span, even though the prediction was weak, indicating that the relationship between these 2 variables is complex and may be influenced by other factors not captured in this analysis. These results indicate several critical updates of the impact of the COVID-19 on spoken language processing and verbal working memory.

### SRP Results

Our findings reveal a surprising impact of COVID-19 on the spoken language of young adults, challenging the prevailing assumption that only infants and children in the developmental stage are susceptible to such effects. Although prior research has predominantly linked COVID-19 with developmental language disorders or delays, particularly in infants and children [36-39], this study underscores the unique vulnerability of healthy young adults to disruptions in story retelling as a result of COVID-19 infection. While children in the developmental stage are generally considered more vulnerable to language impairments, young adults typically exhibit stable story retelling abilities. However, this study suggests that COVID-19 may be associated with disruptions in these abilities. The duration and trajectory of such effects remain unclear, particularly in individuals experiencing post–COVID-19 condition. Future longitudinal research is needed to clarify whether and how story retelling performance may change over time. Additionally, this study suggests that story retelling may be especially sensitive in detecting the cognitive impact of COVID-19. This heightened sensitivity may be attributed to the complexity of the language system, wherein discourse-level processing involves multiple cognitive and linguistic components. In this context, the SRP, which heavily relies on auditory comprehension and various memory functions [16,29,40-42], emerges as a particularly sensitive tool. Both auditory comprehension and memory have been identified as vulnerable indicators of disruptions in language processing abilities [43,44]. Given the complexity of language processing assessed by the SRP, it holds promise as an invaluable tool for detecting the nuanced effects of COVID-19 on story retelling in young adults.

### Verbal Working Memory Findings

Conversely, we observed no significant impact of COVID-19 on verbal working memory. These findings contrast with those reported in previous literature, which has documented negative effects of COVID-19 on working memory [45]. One possible explanation for this discrepancy is the age group of the participants in this study. According to recent research [45], the detrimental effects of COVID-19 on working memory function are primarily observed in adults aged 25 years and older. In contrast, the average age of the participants in the COVID-19 group in this study was 21.37 years, with the non–COVID-19 group also consisting of individuals in a similar age range. This age-related difference may account for the absence of significant working memory decline in the younger cohort in this study. Another potential factor is that none of the participants had been hospitalized for COVID-19, and most reported only mild symptoms. It is also possible that some were asymptomatic. This limited severity may help explain the absence of significant group differences in working memory performance. Moreover, this study did not collect data on whether the COVID-19 group experienced brain fog or post–COVID-19 condition, as data collection took place shortly after the acute phase of COVID-19 infection. It is possible that participants did not exhibit symptoms of post–COVID-19 condition related to cognitive decline or other persistent neuropsychological impairments. Given these factors, further research is needed to examine verbal working memory in individuals experiencing symptoms of post–COVID-19 condition, as this cohort may exhibit different cognitive outcomes.

### Results of Regression Modeling

Regression modeling revealed that the story retelling performance was predicted by the verbal working memory test, specifically the Alphabet Span task. This association between SRP and verbal working memory was previously identified in a study involving stroke survivors with aphasia [16]. Revisiting these results with a narrowed focus on individuals exhibiting symptoms of post–COVID-19 condition is crucial to further clarify this predictive relationship further.

### Limitations

As discussed earlier, a notable limitation of this study is the absence of information regarding whether participants with a history of COVID-19 had experienced post–COVID-19 condition. This gap in data collection stems from the timing of the study, which occurred shortly after the onset of COVID-19. In addition, this study was conducted in a virtual testing environment, which may have introduced variability in participants’ attention, environmental distractions, or audio quality, potentially affecting performance. Another limitation is the possibility of selection bias, as participants volunteered to complete the study remotely and may not fully represent the broader young adult population. Furthermore, the regression analyses yielded relatively low predictive power, suggesting that while certain predictors showed statistical associations, they may not account for a substantial proportion of variance in story retelling performance.

### Conclusion

This study identified differences in spoken story retelling performance between individuals with and those without a history of COVID-19. Additionally, story retelling performance was predicted by the Alphabet Span Test, a measure of verbal working memory. These findings suggest that story retelling may be sensitive to subtle post–COVID-19 cognitive-linguistic changes in young adults. This has potential clinical implications for early cognitive-linguistic screening and targeted support interventions, particularly in educational and clinical settings.

## Abbreviations

IUs/min: information units per minute
SRP: Story Retelling Procedure
